# Boric Acid Diminishes Sciatic Nerve Injury-Induced Apoptosis, Oxidative Stress, and Pain via The Block of TRPV1 Channel in Mice

**DOI:** 10.1007/s12011-025-04698-8

**Published:** 2025-06-28

**Authors:** Kemal Ertilav, Mustafa Nazıroğlu

**Affiliations:** 1 Department of Neurosurgery, School of Medicine, University of Suleyman Demirel, Isparta, Türkiye; 2 Center of Neuroscience Research (NOROBAM), University of Suleyman Demirel, Isparta, Türkiye; 3 BSN Health, Analyses, Innov., Consult., Organization, Agricul. and Industry Ltd., Isparta, Türkiye; 4 Department of Biophysics, School of Medicine, University of Suleyman Demirel, Isparta, Türkiye

**Keywords:** Apoptosis, Boric acid, Sciatic nerve injury, Mitochondrial oxidative stress, TRPV1

## Abstract

The main actors of sciatic nerve injury (SNI) are pain, apoptosis, excessive reactive oxygen species (ROS), and Ca^2+^ entry. However, the role of antioxidant and antiapoptotic boric acid (BoA) through TRPV1 inhibition on the actors in SNI-induced mice has not yet been elucidated. We investigated whether BoA protected the SNI actors in mice undergoing SNI. The thirty-two mice were divided into four groups: Control, BoA, SNI, and SNI + BoA. For four weeks following SNI induction, the BoA and SNI + BoA received 100 mg/kg BoA intraperitoneally. The SNI group, but not the BoA or BoA + SNI groups, indicated increases in TRPV1 current density and Ca^2+^ concentration induced by the TRPV1 agonist (capsaicin). The SNI + BoA group had a reduction in the increases of pain intensity (threshold of paw withdrawal and delay of thermal paw withdrawal) induced by SNI. In the brain, blood, and sciatic nerve of the SNI group, BoA and TRPV1 antagonist (capsazepine) treatments reduced the increases of mitochondrial membrane dysfunction, apoptosis, caspases (-3, -8, and -9), lipid peroxidation, mitochondrial, and intracellular ROS caused by SNI through upregulation of cell viability and antioxidants (vitamin A, vitamin E, β-carotene, glutathione, and glutathione peroxidase). In conclusion, BoA therapy reduced the rise in mitochondrial ROS, apoptosis, and Ca^2+^ entry in the sciatic nerve via inhibiting TRPV1. Therefore, the BoA may be a useful novel treatment through modulation of TRPV1 for oxidative stress, apoptosis, and pain produced by SNI.

## Introduction

Crush injuries rank highest among other injury types, and peripheral nerve damage might cause lifelong disability [1]. People may have significant functional impairment and a decline in their quality of life as a result of sensory and motor function loss induced by physical damage to peripheral nerves [2]. Ca^2+^ influx-induced apoptosis, mitochondrial (mROS), and intracellular (iROS) reactive oxygen species play crucial roles in the etiology of motor function loss and sensory deficiency in sciatic nerve injury (SNI) [3–5]. Neurotrophins, extracellular matrix molecules, and electrical stimulation are some of the therapeutic strategies that have been developed in the past ten years to promote nerve regeneration by inhibiting oxidative stress and Ca^2+^ influx [6–10]. Regretfully, none of these methods can guarantee a full recovery of function; hence their usage is restricted. New approaches are therefore required to improve axonal regeneration, encourage remyelination, and restore neuronal function. Many different kinds of drugs have been utilized in experiments by blocking the Ca^2+^ influx, mROS, and apoptosis in rodent crush injury models, such as voltage-gated calcium channel blockers, antioxidant plants, and trace elements [4, 7, 11–13]. Among other therapeutic strategies, pharmacotherapy is a promising approach to neurorehabilitation, with the exploration of potential natural products that can improve the efficacy of nerve regeneration attracting considerable research interest [7, 12].

Increased intracellular concentration of free Ca^2+^ ([Ca^2+^]_i_) causes an increase in mitochondrial membrane dysfunction (MMD) in neurons. Three primary pathways are subsequently stimulated by its increase: (1) an increase in the production of mROS and iROS; (2) stimulation of Ca^2+^ permeable cation channels; and (3) the generation of apoptosis and neuron death through the stimulation of active caspases −3 (CASP3), −8 (CASP8), and −9 (CASP9) [3, 14, 15]. Capsaicin (CAPS) from hot chili peppers and mROS products from oxidative stress are the main stimulators of transient receptor potential (TRP) vanilloid 1 (TRPV1), while capsazepine (CAPZ) blocks it [16, 17]. The sciatic nerve has a high expression level of TRPV1 [5]. In rats with SNI, TRPV1 activation increased the amounts of caspase and apoptosis in the sciatic nerve [18]. According to the results of the recent studies [5, 19], the dorsal root ganglion and sciatic nerve of rats with SNI produce more mROS and iROS when SNI-mediated Ca^2+^ signals via TRPV1 activation trigger the apoptotic and pain signaling pathway and increase mitochondrial Ca^2+^ accumulation. Therefore, TRPV1 blockage may be employed as a therapeutic drug in SNI treatments to reduce apoptosis and sciatic nerve death.

Boron is a trace element that occurs naturally as boric acid (BoA), borax, coleminate, and borona trocalcite. BoA plays a role in physiological processes such as wound healing, enzyme reactions, and cell membrane function [20, 21]. The beneficial effects of BoA on SNI damage of experimental animals [22] and cisplatin-induced neuropathy were recently documented [23]. By upregulating the antioxidants [glutathione (GSH) peroxidase (GSHPx) and reduced GSH] but downregulating the caspases, lipid peroxidation (LPx), iROS and mROS formation in the kidney, BoA acted antioxidant and antiapoptotic functions [24, 25]. However, the antioxidant and antiapoptotic properties of BoA through TRPV1 channel blockage in the sciatic nerve of SNI-induced mice were not investigated.

Designing alternative therapeutics requires an understanding of how BoA inhibits TRPV1 activation in the brain and sciatic nerve of SNI-induced animals; hence modulating SNI-caused Ca^2+^ influx, mROS production, and apoptosis induction. We propose that the protective function of BoA through TRPV1 suppression could help treat SNI and pain in mice.

## Materials and Methods

### Mice

We purchased thirty-two male C57BL/6j mice, aged twelve to fourteen weeks, weighing twenty to twenty-five grams, from the Center of Experimental Animal, University of Burdur Mehmet Akif (MAKU), in Türkiye. They receive ad libitum access to commercial feed mixture from Kortkutelim Animal and Human Food Inc. (Korkuteli, Antalya, Türkiye) and free tap water. Each experiment was evaluated and authorized by the Local Ethical Committee of Animal Experiments in MAKU on March 13, 2024 (The number of meetings: 121; Decision Number: 1279), in accordance with the MAKU rules.

### Experimental Study Groups

The mice were split into four equal groups: control (CNT), BoA, SNI, and SNI + BoA. For four weeks, the CNT group received physiological saline intraperitoneally. The BoA group was given 100 mg/kg/day of BoA intraperitoneally for four weeks [25, 26]. The surgical technique was applied to mice in the SNI group and their right leg was ligatured according to the method of Bennett and Xie [27]. The SNI groups intraperitoneally received physiological saline for four weeks. After three days following the induction of SNI, the mice in the SNI + BoA group were given BoA supplements for four weeks, but they were exposed to the identical injury as the SNI group.

### Assessment of the Withdrawal Threshold for the Hind Paw

Mice in the four groups were tested for pain sensitivity using von Frey monofilaments (20PC Aesthe, Muromachi Kikai Ltd., Tokyo, Japan) and a hot plate (Variomag, Langenselbold, Germany). As previously described [5], we determined the latency of thermal paw withdrawal (hot plate) and threshold of paw withdrawal (von Frey) of mice both before and after SNI induction and BoA treatment. With a 20-s cutoff time, the delay (latency) to the first paw reaction (second) was measured. Either a foot shake or a paw lick was considered a paw response.

### SNI Induction and Red Blood Cell, Plasma, Brain, and Sciatic Nerve Preparation

**Fig. 1:**
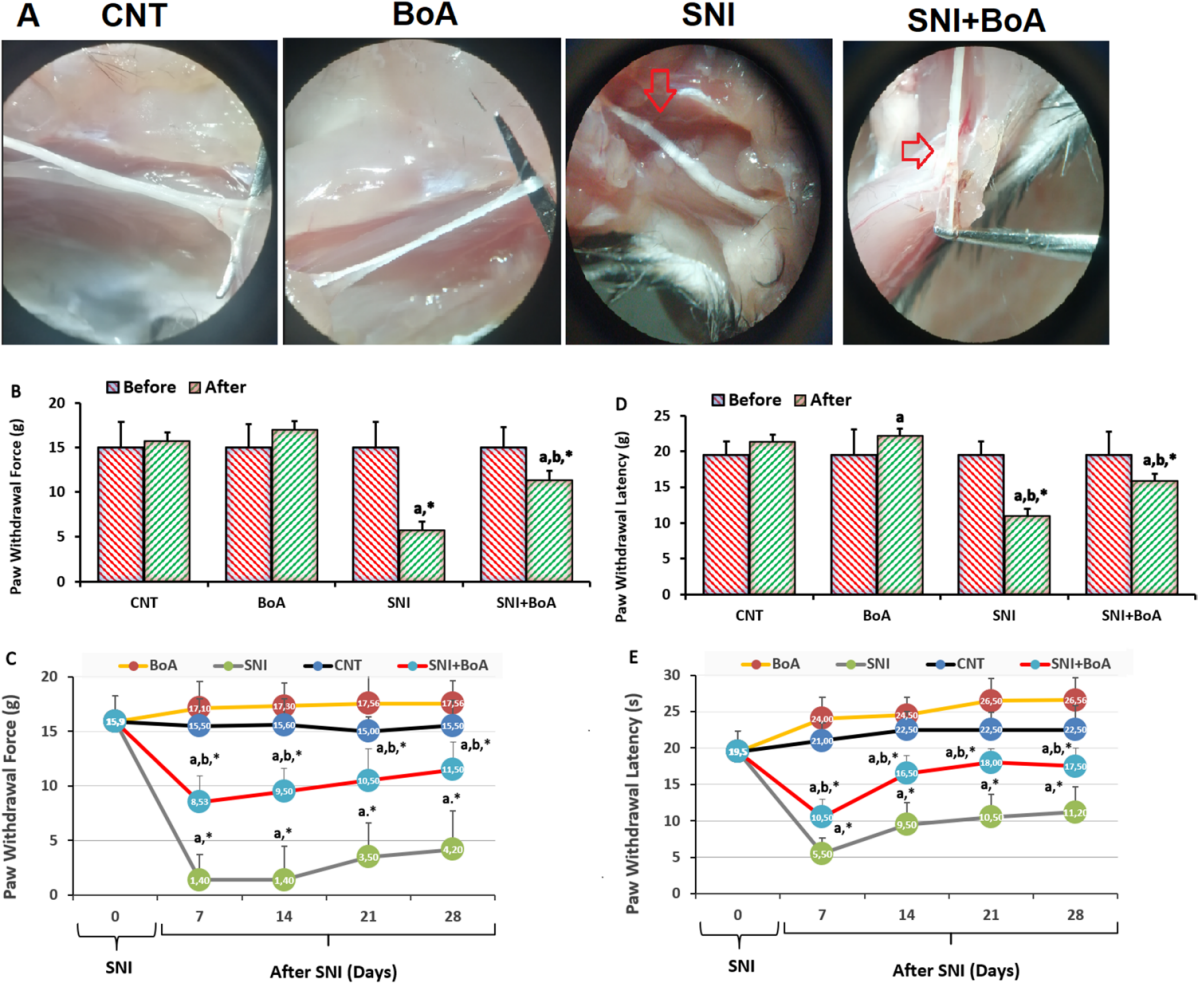
Four-week 100 mg/kg injection of BoA decreased the increase in pain intensity induced by SNI. (mean ± SD; N = 8). **A**. After four weeks, pictures of the sciatic nerves in each of the four groups. In order to quantify paw withdrawal force (g) and paw withdrawal delay (second), the von Frey (**B** and **C**) and hot plate (**D** and **E**) were utilized. (^a^p < 0.05 in comparison to CNT and BoA. ^b^p < 0.05 to SNI.^*^p < 0.05 to zero day)

After induction of xylazine and ketamine anesthesia, the right hind common sciatic nerve of the paw was bluntly dissected through the biceps femoris to reveal the middle of the thigh [5, 14]. A hemostatic sterile clamp was utilized to treat crush of sciatic nerve. For thirty seconds in all, the sciatic nerve was crushed. After a 2.0 suture was used to close the wound, the rats were left in the postoperative room to heal (Fig. 1A).

After four weeks, samples of sciatic nerves from the right leg were carefully dissected. The DMEM cell culture medium in which the neurons were incubated. The sciatic nerve was treated with 0.28 ml collagenase IV and 25,000 units/ml tyripsin for 45 min at 37 ºC after the connective tissue was removed. The sciatic nerve suspension of media was prepared by centrifuging it at 1,500 g after dissociating it with a sterile syringe. The neurons were then extracted for patch-clamp analysis and plate reader [5]. The imaging analyses of a laser scan confocal microscope with 20 × 0.8 objective (LSCM-800) (LSM 800, Carl Zeiss AG, Oberkochen, Germany) was performed on whole sciatic nerve samples.

For the LPx and antioxidant analysis, there weren't enough sciatic nerve samples. Hence, red blood cells (RBC), cerebral cortex, and plasma samples were used to assay the antioxidant and LPx concentrations. An ultrasonic homogenizer (HD2200, Bandelin, Berlin, Germany) was used to prepare the cerebral cortical homogenates [15]. Using centrifugation (500 g for 5 min), plasma samples were extracted from the whole blood tube using the anticoagulant (disodium EDTA). The RBC at the bottom of the tube was washed with physiological saline for generating hemolysate.

### Measurement of [Ca^2+^]_i_ Changes in the Whole Sciatic Nerve

The LSCM-800 was used to record the green pictures of the whole sciatic nerve in the bottom glass plates (Cat # P35G-1.5–14-C, Mattek, Ashland, MA, USA) in order to measure changes in [Ca^2+^]_i_ fluorescence intensity. Before being stimulated by an argon laser at 488 nm, the whole sciatic nerve samples were dyed for 60—90 min using 1 µM Fluo −3/AM dye (Cat # ab145254, Abcam Inc., Cambridge, UK) [15]. Changes in fluorescence intensity in collected green images were measured using a customized application (ZEN, blue edition). The [Ca^2+^]_i_ fluorescence intensity data were displayed using the arbitrary unit (a.u.).

### Patch-Clamp Recordings

The whole-cell patch-clamp recording techniques was used in the sciatic nerve for the detection of TRPV1 currents [5]. The details of extracellular and intracellular buffers were indicated in a previous study [5]. The Na^+^-free extracellular solution was prepared by adding 150 mM N-methyl-D-glucamine (NMDG^+^). Following the activation of the whole cell configuration, we waited until the currents decreased to their baseline (control) levels. We conducted the CAPZ and NMDG^+^ treatments to block the TRPV1 currents because we noticed an inward current in the neurons following the application of CAPZ or NMDG^+^. To measure the currents, the EPC10 amplifier (HEKA, Stuttgart, Germany) was utilized. TRPV1 was stimulated in the sciatic nerve by 10 µM CAPS and inhibited by 100 µM CAPZ. For the presentation of data, the current intensity (pA/pF) value was used.

### Evaluations of Apoptosis, Cell Viability, and Caspase Levels

A density of 2 × 10^3^ sciatic nerve per milliliter was seeded in six-well plates with 100 µl of MTT (5 mg/ml) in phosphate buffer solution. Following that, the white plates were incubated for four hours at 37 °C. The 500 µl of DMSO was added in an attempt to dissolve the formazan crystals. After a minute of gentle shaking, the absorbance of six-well plate was measured at 492 nm using a UV-1800 spectrophotometer (Shimadzu, Kyoto, Japan). An Infinite 200 PRO microplate reader (Tecan Inc., Männedorf, Switzerland) was used to quantify the quantity of sciatic nerve apoptosis using the commercially available APOPercentage apoptosis evaluation kit from Biocolor Ltd. (Cat # A1000, County Antrim, UK) [15, 28].

The fluorogenic substrates of CASP3 (Ac-DEVD-AMC), CASP8 (Ac-IETD-AFC), and CASP9 (Ac-LEHD-AFC) (Bachem AG., Bubendorf, Switzerland) were mostly cleaved by active, CASP8, and CASP9 to the AMC and AFCs, respectively, after being incubated with apoptotic cell lysates in black plates. Free AMC and AFC cleavage levels were determined using the Infinite 200 PRO microplate reader at the excitation (360–400 nm) and emission (460–505 nm) wavelengths [29, 30].

Percentage (% of control) presentations of sciatic nerve viability, apoptosis, CASP3, CASP8, and CASP9 were determined utilizing the optical density/fluorescence intensity measurements.

### The Detection of Percentage of Propidium Iodide (PI)-Positive Cell in the Whole Sciatic Nerve

Hoechst 33,342 (Cat # H3570, ThermoFisher Scientific) and PI (Cat # P1304MP, ThermoFisher Scientific) were used to calculate the cell death percentage. The neurons in the dish media were labeled with PI (1.5 mM) and Hoechst 33342 (8.1 mM). After a 20-min incubation time, the Axiocam 702 fluorescent camera and the fluorescent microscope (Zeiss Axio Observer.Z1/7) were used to take pictures of the blue (Hoechst), red (PI), 2.5D, and bright field (BF). To calculate the percentage of PI-positive cells in the Axiocam 702 camera evaluation, every single cell in the gathered cells was manually counted.

### The Detection of Mitochondrial Membrane Dysfunction (MMD), iROS, and mROS in the Whole Sciatic Nerve

The MMD as a reduction in the membrane potential of the mitochondria in whole sciatic nerve stained with JC1 (Cat # T3168, Thermo Fischer) was detected in the LSCM-800 by using a diode argon laser stimulation at 488 nm [31]. The manufacturer (Thermo Fischer) instructed that as mitochondrial membrane hyperpolarization increases, the mitochondrial membrane potential decreases. This was associated with a decrease in the JC1 (J-aggregates/J-monomers) ratio and an increase in orange color. As a result, the orange JC1 color of the neurons was recorded in order to measure the MMD.

A mROS indicator for live-cell imaging, MitoSOX Red reagent of ThermoFischer (Cat # M36008), was used to measure the amount of mROS. The neurons from the experimental groups were incubated for 15–20 min at body temperature (37 °C) in the dark after MitoSOX (5 ⁭µM) was added. The diode argon laser at 561 nm was used stimulation of MitoSOX Red. The red images were recorded with the LSCM-800.

Diacetyldichlorofluorescein (DCFH-DA) of Abcam (Cat # ab113851, Istanbul, Türkiye), a nonfluorescent dye, easily penetrates cell membranes. When this probe is oxidized via hydrogen peroxide generation, it produces dichlorofluorescein (DCF), and the amount of iROS produced is directly associated with the fluorescence of DCF [32]. A diode argon laser at 488 nm in the LSCM-800 was then used to record the green DCF images.

Using the ZEN program, the resulting red (MitoSOX), orange (JC1), and green (DCF) fluorescence intensity changes in the images were expressed as a.u. [28].

### The Analyses of GSH, GSHPx, LPx, and Antioxidant Vitamin Concentrations in Brain, Plasma, and RBC

In the brain homogenate and RBC hemolysate, the optical density changes of GSHPx (at 412 nm) [33], GSH (at 412 nm) [34], LPx (at 532 nm), and total protein [35] were manually assessed using a UV-1800 spectrophotometer. A comprehensive analysis was presented in earlier studies [15, 28]. However, the GSHPx activity was evaluated in IU/gram protein, even though the brain and RBC levels of LPx and GSH are expressed in µmol/gram protein.

Vitamin A, β-carotene, and vitamin E levels in the brain and plasma were manually measured using the UV-1800 spectrophotometer [36, 37]. For retinol, the wavelengths were set at 325 nm, 450 nm for β-carotene, and 532 nm for α-tocopherol. Vitamin A, β-carotene, and vitamin E were measured by dissolving the standard solutions of all-trans retinol, β-carotene, and α-tocopherol in hexane. Although vitamin concentrations were expressed in plasma as µ⁭mol/l, they were reported as ⁭µmol/g tissue in brain tissue.

### The Analyses of Statistics

After calculating the mean ± standard deviation (SD) values from the mean data, the SPSS program (Version 22.0, Chicago, IL, USA) was used to evaluate the statistical significance of each group using Fisher's least significant difference (LSD) test. Using the one-way analysis of variance (ANOVA), the statistical significance of groups (p < 0.05) was evaluated.

## Results

### SNI-Induced Elevations in Mechanical and Thermal Hyperalgesia Were Reduced by BoA Injections

For 28 days after the induction of SNI in the von Frey (Figs. 1B and 1 C) and hot plate (Figs. 1D and 1E), the withdrawal force of mechanical hind paw (von Frey) and withdrawal latency of heat (hot plate), respectively, were evaluated and indicated a progressive rise. The withdrawal force of mechanical hind paw and withdrawal latency of heat were thereby reduced in the BoA and SNI + BoA (p < 0.05) as a result of the BoA treatments as opposed to the SNI alone.

### SNI-induced TRPV1 Activation Caused a Rise in [Ca^2+^]_i_), But a BoA Injection Induced a Decrease

**Fig. 2:**
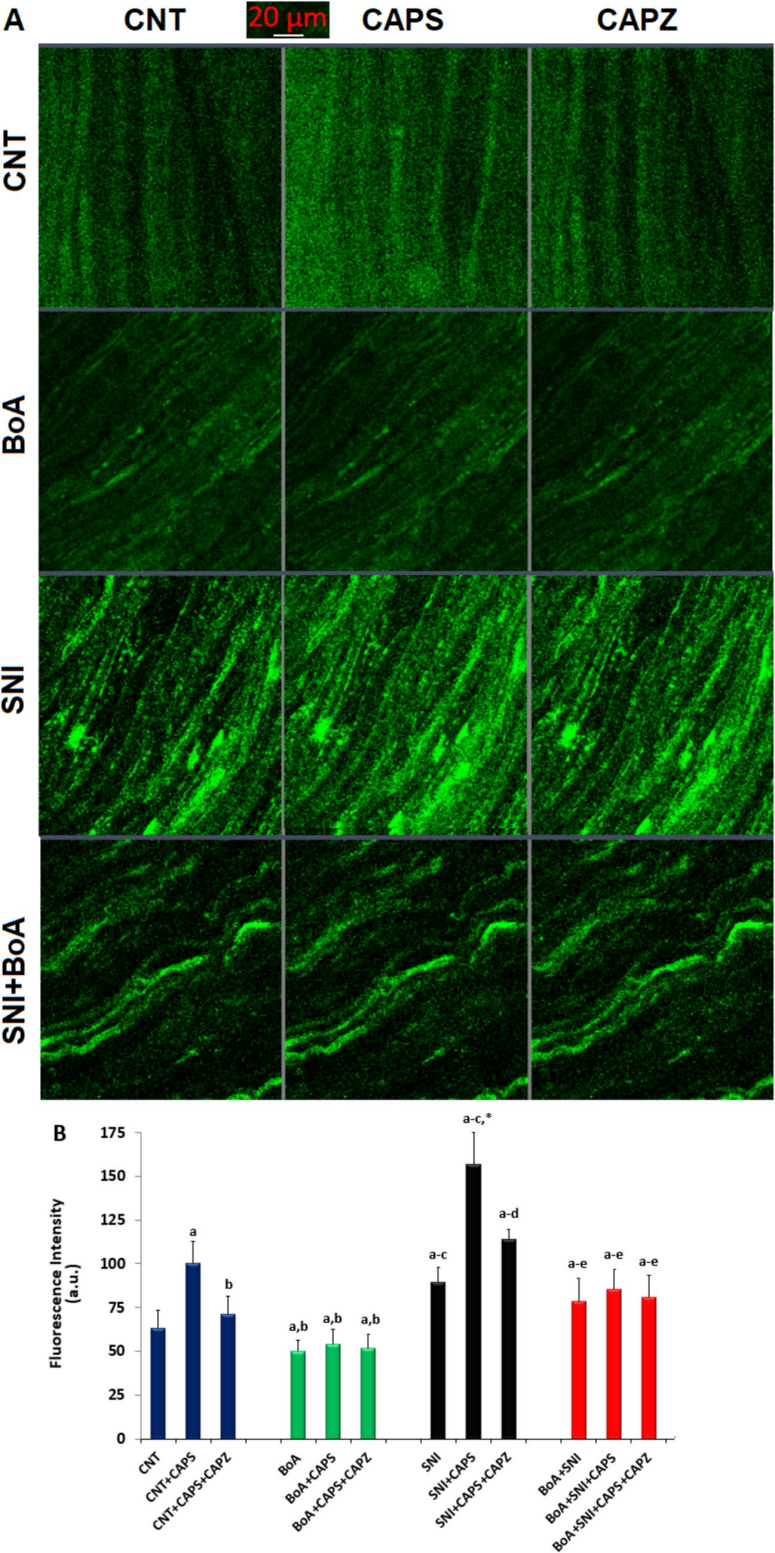
BoA (100 mg/kg) was injected into the mouse for four weeks, which blocked the TRPV1 channel and decreased the rise in [Ca^2+^]_i_ levels induced by SNI. The mean ± SD for N = 8. **A**. The LSCM-800 was used to capture the green pictures from each of the four groups after the entire sciatic nerve had been labeled with Fluo −3/AM (1 µM) for 60 to 90 min. **B**. The addition of CAPS (10 ⁭µM) and CAPZ (100 ⁭µM) causes a change in the [Ca^2+^]_i_ fluorescence intensity in each of the four groups. a.u. is an arbitrary unit. There is a 20 µm scale bar. (^a^p < 0.05 in comparison to*.* CNT, ^b^p < 0.05 to CNT + CAPS. ^c^p < 0.05 to BoA, BoA + CAPS, and BoA + CAPS + CAPZ, *p < 0.05 to SNI, ^d^p < 0.05 to SNI + CAPS, ^e^p < 0.05 to SNI + CAPS + CAPZ.)

The SNI group possessed higher [Ca^2+^]_i_ in the whole sciatic nerve than the CNT and BoA groups (Figs. 2A and 2B). Nevertheless, the CAPS (10 µM)-induced increase of [Ca^2+^]_i_ in neurons was considerably (p < 0.05) decreased by treatment with a TRPV1 antagonist (100 ⁭⁭µM CAPZ). Without the SNI, there was no increase in the [Ca^2+^]_i_ concentration in the BoA group (p > 0.05). But in the SNI + BoA neurons, the BoA injections dramatically decreased the rise in [Ca^2+^]_i_ concentration (p < 0.05).

### The Rise in TRPV1 Current Density in the Sciatic Nerve Induced By SNI Was Reduced by the BoA

**Fig. 3:**
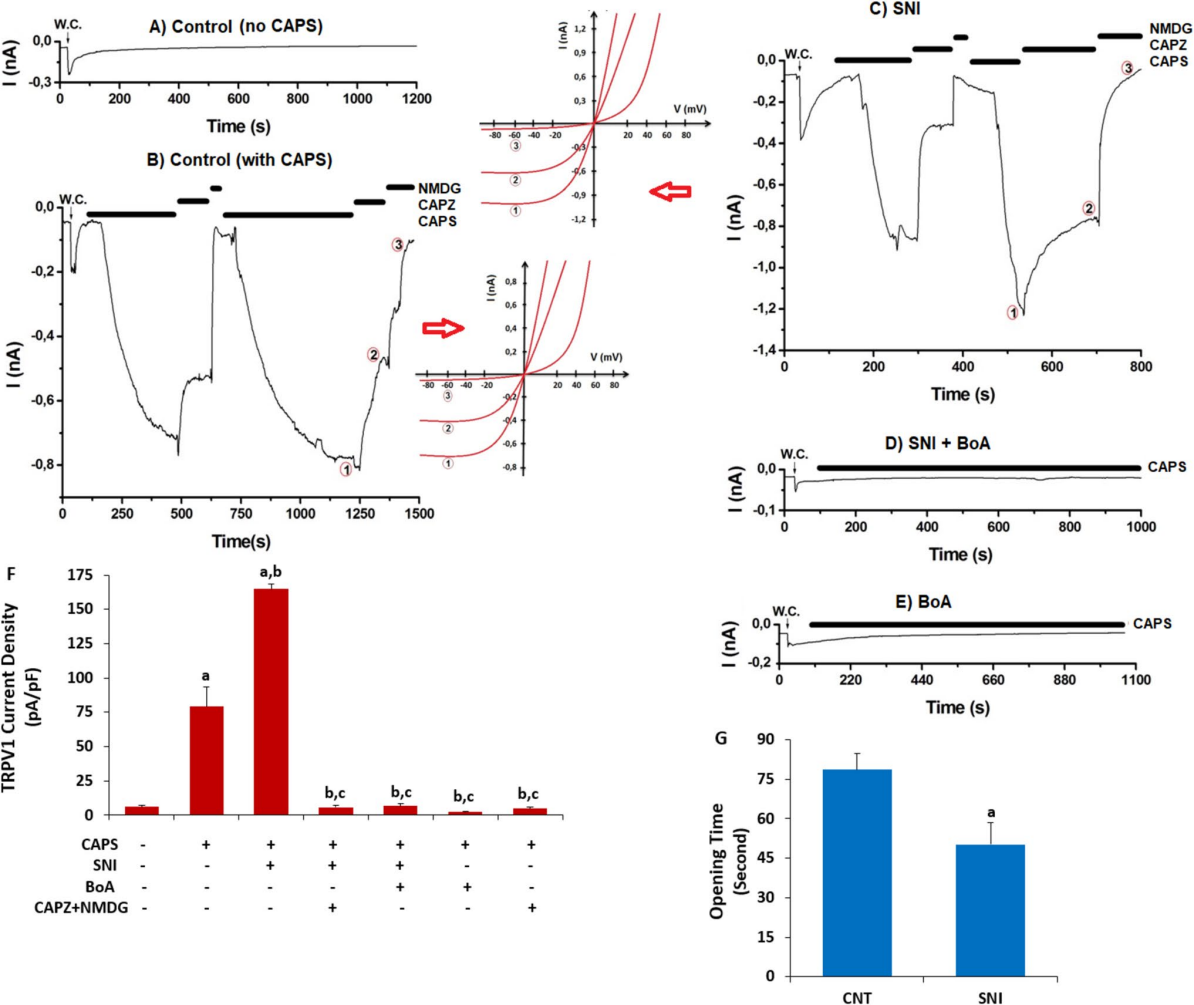
The increase in current density of TRPV1 in the sciatic nerve induced by SNI was reduced by the four-week 100 mg/kg BoA injections. (N = 8; mean ± SD). CAPS (10 ⁭µM) activated TRPV1, whereas CAPZ (100 µM) suppressed it. A. CNT control (no CAPS agonist). **B**. CNT + CAPS that includes CAPZ/NMDG group. **C**. The SNI group that includes CAPS and CAPZ. Figures B and C demonstrate the correlation between current (I) and voltage (V), respectively. I/V lines in (1) CAPS, (2) NMDG, and (3) CAPZ. **D**. BoA with SNI. **E**. BoA. **F**. The mean density of TRPV1 current. **G**. TRPV1 opening times of CNT and SNI groups as second. (^a^p < 0.05 in comparison to CNT, ^b^p < 0.05 to CNT + CAPS, and ^c^p < 0.05 to SNI + CAPS)

There was a limited current as pA/pF in the CNT sciatic nerve that were not stimulated by CAPS (Fig. 3A). However, the TRPV1 current in the CNT neurons was triggered by the addition of extracellular CAPS (10 μM) (Fig. 3B). The CNT + CAPS group had higher mean TRPV1 current densities (79.24 pA/pF) than the CNT group (6.18 pA/pF) (Figs. 3A and 3 F) (p < 0.05). The mean current densities of TRPV1 were higher in the SNI + CAPS group (165.33 pA/pF) than in the CNT + CAPS group (p < 0.05) (Fig. 3C). Both the CNT + CAPS + CAPZ + NMDG (4.70 pA/pF) and SNI + CAPS + CAPZ + NMDG (5.60 pA/pF) groups had lower TRPV1 current densities (p < 0.05) than the CNT + CAPS and SNI + CAPS groups. We observed limited TRPV1 currents when BoA was treated with SNI (6.53 pA/pF; Fig. 3D) or without SNI (2.59 pA/pF; Fig. 3E). It required 78.60 s to observe the CAP-induced TRPV1 currents in the control group and 50.20 s to obtain them in the SNI groups (Fig. 3G). Therefore, SNI was employed to speed up TRPV1 stimulation (p < 0.05).

### The SNI-Mediated Increases of Apoptotic and Sciatic Nerve Death Indicators Were Reduced by BoA

**Fig. 4:**
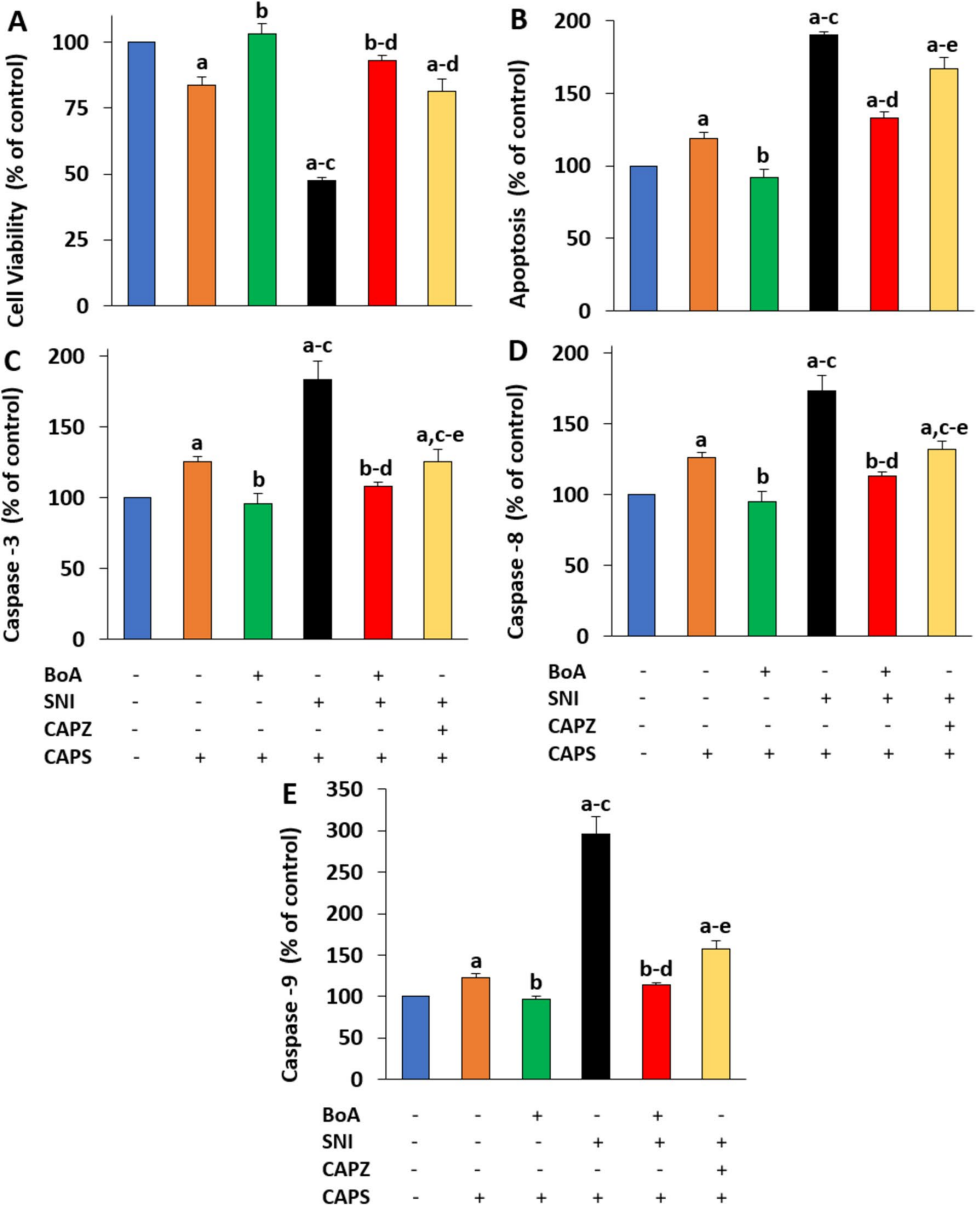
The increase in apoptosis and caspases induced by SNI was prevented by a four-week injection of BoA (100 mg/kg). (N = 8; mean ± SD). Using an MTT test in combination with a commercial kit (APOpercantage), UV-1800 spectrophotometer, and a plate reader (Infinite PRO 200), the quantity of cell viability (**A**) and apoptosis (**B**) in the sciatic nerve was assessed. In the plate reader, the caspase substrates were used to measure the amounts of caspases −3 (**C**), −8 (**D**), and −9 (**E**). (^a^p < 0.05 in comparison to CNT without CAPS, ^a^p < 0.05 to CNT with CAPS, ^b^p < 0.05 to CNT without CAPS ^c^p < 0.05 to BoA + CAPS, ^d^p < 0.05 to SNI + CAPS, and ^e^p < 0.05 to SNI + BoA + CAPS)

The neuron viability percentages of SNI were lower (p < 0.05) than those of the CNT and BoA (Fig. 4A). But in the SNI + BoA and SNI + CAPZ groups, the addition of CAPZ and BoA also significantly (p < 0.05) increased neuron vitality.The levels of apoptosis (Fig. 4B), CASP3 (Fig. 4C), CASP8 (Fig. 4D), CASP9 (Fig. 4E), and in the sciatic nerve were all stimulated by SNI. Comparing the SNI group to the CNT and BoA, the SNI group displayed greater levels of apoptosis, CASP3, CASP8, and CASP9. SNI plus BoA and SNI plus CAPZ had less significance than SNI alone (p < 0.05).

**Fig. 5:**
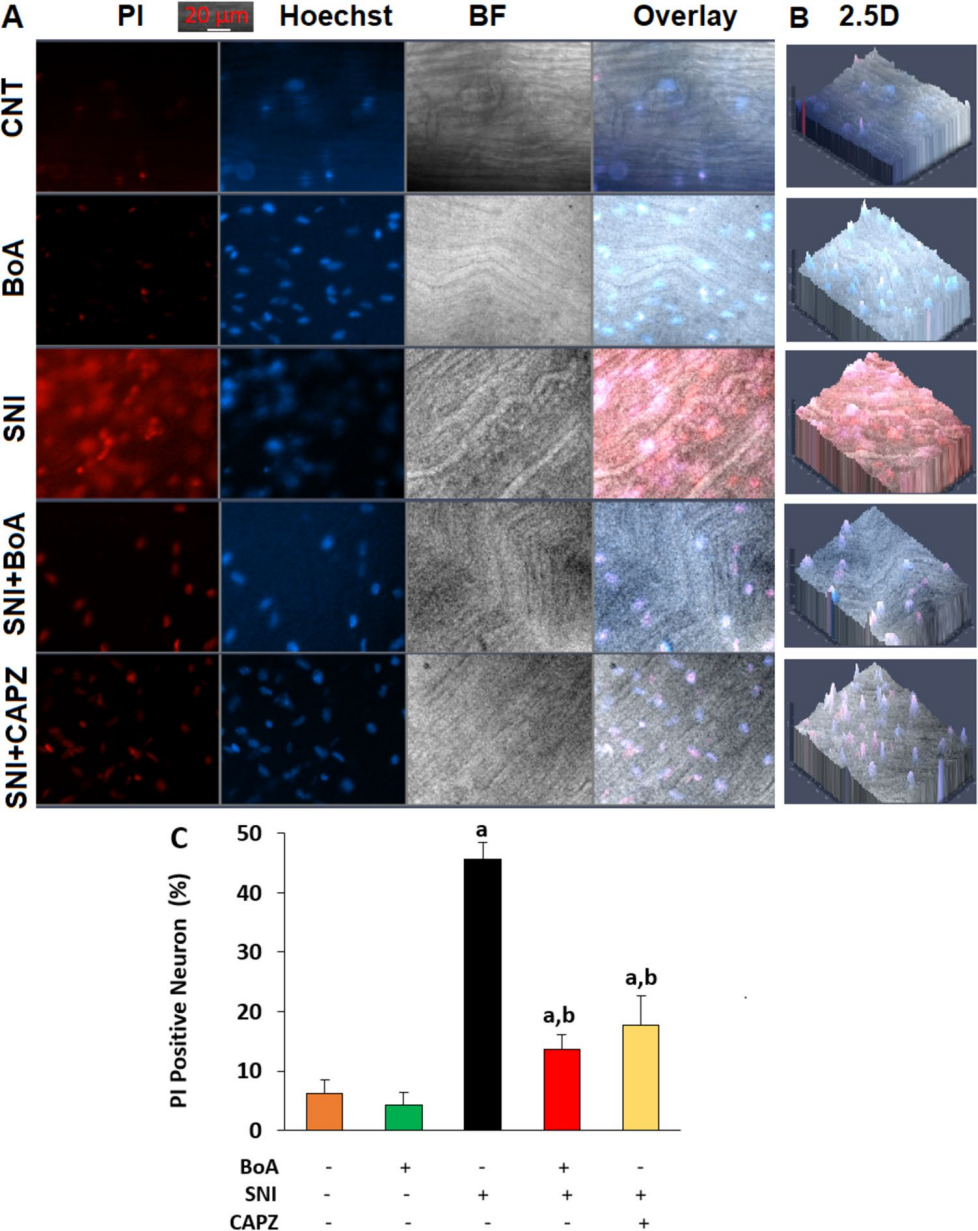
An injection of BoA (100 mg/kg) decreased the increase in the percentage of death neurons caused by SNI. (The mean for N = 8 ± SD). **A**. Images of the whole sciatic nerve in dead (PI), live (Hoechst 33342), bright field (BF), and overlay. **B**. The 2.5D images of whole sciatic nerve. The sciatic nerve in the SNI plus CAPZ group was incubated with CAPZ (100 ⁭⁭µM) for an hour following its removal from the SNI groups. At 20 μm, we kept the scale bar in the images. The Axiocam 702 camera was used to capture the pictures. **C**. The average percentages of neurons with PI. (^a^p < 0.05 in comparison to CNT and BoA. ^b^p < 0.05 to SNI)

In comparison to the CNT and BoA groups, SNI induction increased sciatic nerve death (PI positive cell percentage) in the SNI group (p < 0.05), as evidenced by the red/blue (PI/Hoechst), bright field (BF), overlay (Fig. 5A), and 2.5D images (Fig. 5B). However, the sciatic nerve-death action of SNI was reduced (p < 0.05) in the SNI plus BoA and SNI plus CAPZ groups by the CAPZ and BoA treatments (Fig. 5C).

### The Rise in Oxidative Stress and Mitochondrial Dysfunction Induced By SNI Was Decreased by the Injections of BoA

**Fig. 6:**
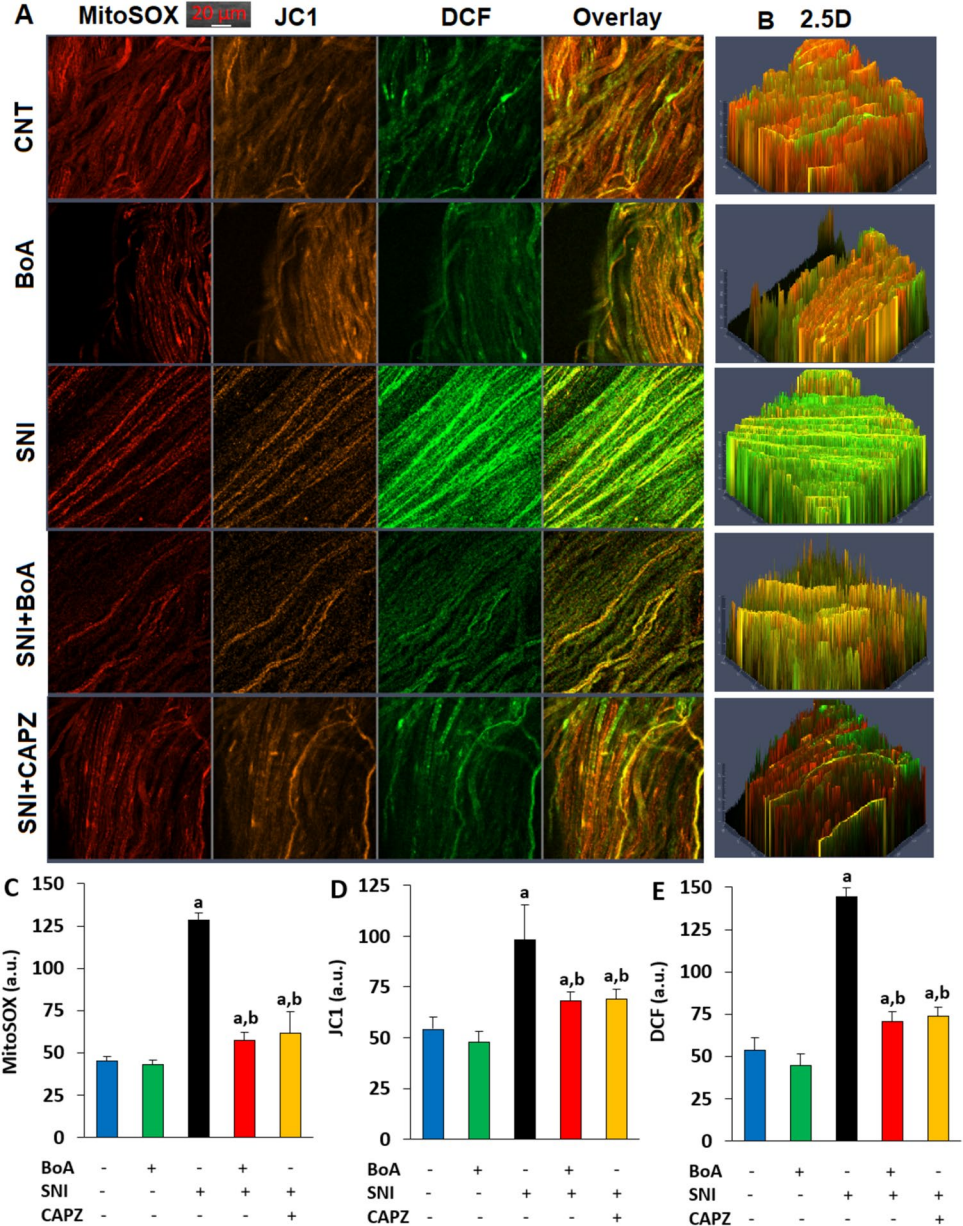
Injections of BoA (100 mg/kg) over a four-week period decreased the increased oxidative stress and mitochondrial dysfunction of the sciatic nerve induced by SNI. The mean ± SD for N = 8. **A**. With the LSCM-800, color pictures of mROS/MitoSOX (red), MMD/JC1 (orange), iROS/DCF (green), overlay, and 2.5D (**B**) were taken. For MitoSOX (**C**), JC1 **(D**), and DCF (**E**), the mean fluorescence intensity variations are presented in arbitrary units (a.u.). (^a^p < 0.05 in comparison to CNT and BoA. ^b^p < 0.05 to SNI)

mROS/MitoSOX (Figs. 6A and 6C), MMD/JC1 (Figs. 6A and 6D), iROS/DCF (Figs. 6A and 6E), their overlay and 2.5D (Fig. 6B) photos and fluorescence intensity showed that the sciatic nerve in the SNI group increased levels of DCF, JC1, and MitoSOX as compared to the groups of CNT and BoA (p < 0.05). But in the sciatic nerve of the SNI plus BoA and SNI plus CAPZ groups, the amounts were reduced by the CAPZ and BoA treatments (p < 0.05).

### BoA Injections Modulated the SNI-induced Decreased Levels of Vitamin A, β-carotene, Vitamin E, GSH, and GSHPx through Decrease of LPx

In the SNI group, the brain and RBC had a higher LPx concentration than in the CNT and BoA groups, however in the SNI plus BoA group, their concentrations reduced (p < 0.05) (Table 1). GSH, GSHPx, vitamin A, vitamin E, and β-carotene were lower in the brain, plasma, and RBC of the SNI group than in the CNT and BoA without SNI groups (p < 0.05) (Table 1). The administration of BoA, on the other hand, increased their concentrations in the SNI + BoA (p < 0.05).

**Table 1:** Boric acid (BoA) induced positive effects on the levels of antioxidant vitamins, lipid peroxidation (LPx), reduced glutathione (GSH), and glutathione peroxidase (GSHPx) in the brain, red blood cells (RBC), and plasma of mice with sciatic nerve injury (SNI)

	Values	Control (*n* = 8)	BoA (*n* = 8)	SNI (*n* = 8)	SNI + BoA (*n* = 8)
LPx●	Brain	29.08 ± 4.59	26.12 ± 0.94	39.03 ± 3.27^a^	31.65 ± 2.37^b^
	RBC	20.68 ± 2.34	19.08 ± 2.59	27.46 ± 2.97^a^	20.80 ± 1.55^b^
GSH●	Brain	15.90 ± 1.04	16.30 ± 1.28	9.99 ± 0.50^a^	14.30 ± 0.49^a,b^
	RBC	16.03 ± 2.03	17.10 ± 1.37	12.82 ± 1.18^a^	15.01 ± 0.55^a,b^
GSHPx^Δ^	Brain	24.70 ± 1.17	26.20 ± 2.01	17.10 ± 1.55^a^	23.10 ± 1.65^b^
	RBC	24.60 ± 0.95	23.80 ± 0.61	15.80 ± 1.15^a^	24.00 ± 1.96^b^
α-Tocop	Brain^٭^	20.90 ± 1.14	23.40 ± 1.64	14.10 ± 1.92^a^	20.30 ± 1.81^b^
	Plasma^◊^	22.60 ± 2.63	25.60 ± 1.76	16.50 ± 2.29^a^	21.90 ± 1.70^b^
Retinol	Brain^٭^	3.96 ± 0.43	4.39 ± 0.40	2.91 ± 0.39^a^	3.84 ± 0.54^b^
	Plasma^◊^	5.02 ± 0.80	5.14 ± 0.78	3.57 ± 0.32^a^	4.53 ± 0.29^b^
β-Car	Brain^٭^	1.73 ± 0.24	1.93 ± 0.30	1.28 ± 0.21^a^	1.67 ± 0.20^b^

(*n* = 8 and mean ± SD). (^a^p < 0.05 vs. control and BoA. ^b^p < 0.05 vs. SNI). ●μM/g protein. ^**Δ**^ IU/g protein. ^٭^ μM/g tissue. ^◊^ μM/l tissue

## Discussion

The TRPV1 channel is one of the cation channels whose expression is known to change in response to nerve injury [38, 39]. The permeability of TRPV1 to Ca^2+^, which is implicated in a number of physiological and pathophysiological processes, including pain induction, apoptosis, neuronal viability, and neural recovery signals, increased Ca^2+^ accumulation when it was activated [40]. Sciatic nerve damage has been shown in multiple studies to enhance TRPV1 expression levels in the sciatic nerve and dorsal rot ganglion [12, 41]. Since the TRPV1 channel has been confirmed as a therapeutic target for the control of pain, apoptotic, and oxidant conditions in a variety of diseases and injury states, the development of numerous TRPV1 agonists and antagonists that have entered clinical trials has been promoted [42]. Our earlier research indicated that the antioxidant (*Hypericum perforatum*)-treated group suppressed TRPV1, resulting in lower oxidant levels and apoptotic indicators in the SNI [5]. According to these findings, the antioxidant BoA possesses anti-apoptotic and anti-pain properties by influencing TRPV1 suppression. A follow-up investigation was carried out using an SNI model, assuming that BoA exhibited neuroprotective effects by lowering TRPV1.

For the simultaneous assessment of motor and sensory nerve function following nerve damage, the experimental sciatic nerve model is frequently employed [5, 12]. This research was carried out using verified in vivo models [15, 28]. At the 7th, 14th, 21st, and 28th day following injury, the BoA and SNI + BoA significantly differed from the control group in mechanical and heat pain values in the von Frey and hot plate tests (Fig. 1). The electrophysiological and [Ca^2+^]_i_ experiments results of present investigation. In the tests, we found that the administration of BoA reduced the TRPV1 current densities and [Ca^2+^]_i_ increases that were caused by SNI in the sciatic nerve of the mice. These results consistently showed that BoA has positive effects through downregulation of TRPV1 on the induction of neuronal and functional nerve healing.

Oxidants (LPx, iROS, and mROS) were increased in the frog motor nerve terminal and rat sciatic nerve by an excessive Ca^2+^ influx by TRPV1 activation [15, 43], but antioxidant plant extract therapies reduced the amount of [Ca^2+^]_i_ and oxidants by blocking the TRPV1 in the neurons of laboratory animals with SNI [5, 12]. BoA was found to have positive effects on the SNI damage of experimental animals [22] and cisplatin-induced neuropathy [23]. BoA enhanced antioxidant and antiapoptotic effects by upregulating the antioxidants (GSH and GSHPx) while downregulating the production of LPx, iROS, and mROS in the kidney of rats [24, 25]. In the current study, the LPx, iROS, and mROS levels of sciatic nerve were greater in the SNI group. The suppression of TRPV1 in mice co-treated with BoA resulted in a diminish in the concentrations of mROS, iROS, LPx, and [Ca^2+^]_i_. BoA injections seem to have reduced the rise in oxidant levels and [Ca^2+^]_i_ amount in the sciatic nerve caused by SNI. According to the results, antioxidant injections such as selenium, inosine, and *Hypericum perforatum* decreased the oxidant, TRPA1, TRPM2, and TRPV1 stimulator effects of SNI in the sciatic nerve and dorsal root ganglia of mice and rats with SNI [5, 13, 44, 45].

The elevated [Ca^2+^]_i_ in mitochondria trigger mROS generation and trigger pro-apoptotic signals via CASP3, CASP8, and CASP9 in the brain and neurons, including the sciatic nerve [13, 15, 44–48]. When the pore of mitochondrial permeability transition opens and the amount of [Ca^2+^]_i_ increases due to massive accumulations, the MMD increases [49]. TRPV1 is an oxidative stress-dependent stimulated TRP channel that is activated in the neurons and brain, causing an increase in [Ca^2+^]_i_ [17]. The current data, which show that high [Ca^2+^]_i_ levels arising from TRPV1 activations in SNI eventually damage the mitochondrial membranes, provide support for this theory. The increase in markers of mitochondrial dysfunction (MMD and mROS) and LPx found in sciatic nerve could be attributed to this. In turn, they caused sciatic nerve death and apoptosis by downregulating GSH and GSHPx and upregulating PI-positive cell numbers, CASP3, CASP8, and CASP9 activities [5, 48]. These results collectively highlight the significance of TRPV1-stimulated Ca^2+^ entry in sciatic nerve death or sciatic nerve survival events.

TRP channels, including TRPV1, are most commonly activated by ROS directly through redox modification of the free thiol groups [17, 46]. Antioxidant therapy inhibits the TRPV1 channel by overexpressing GSH and GSHPx, while downregulating GSH and GSHPx promotes the TRPV1 channel [50–52]. Regarding the topic, it was noted that BoA markedly enhanced the experimental depletion of GSH and GSHPx in rat synaptosomes caused by Alzheimer's disease [53]. In the kidney and liver of BoA-treated rats, lipopolysaccharide-induced reductions in GSH and GSHPx were accompanied by increases of CASP/3 and apoptosis [54]. Liver of chronic alcohol-fed rats showed downregulation of LPx, apoptosis, and CASP3 and upregulation of GSH and GSHPx following BoA treatment [55]. Our results showed that BoA protected the sciatic nerve of mice against SNI-induced apoptosis, mitochondrial dysfunction, and a decrease in antioxidant levels. The injections of BoA specifically restored the SNI-mediated decreases of vitamin A, vitamin E and β-carotene, concentrations, as well as TRPV1 activation, CASP3, CASP8, and CASP9, GSH, and GSHPx. More evidence that TRPV1 gate-induced increases in [Ca^2+^]_i_ concentration, MMD, and mROS are the main cause of the upregulations of caspases but decrease of enzymatic and non-enzymatic antioxidants during SNI-induced sciatic nerve death and apoptosis is provided by the current data. However, the treatment by which the BoA injections protected the sciatic nerve was due to an increase of antioxidants and a decrease in caspases.

There are specific weaknesses in the current study. The first step should be to use TRPV1 knockout animals and cell line analyses to corroborate the results. Second, further antioxidants such as vitamin E and N-acetyl cysteine may function as TRPV1 inhibitors in the sciatic nerve. Future studies will evaluate the weakness.

**Fig. 7:**
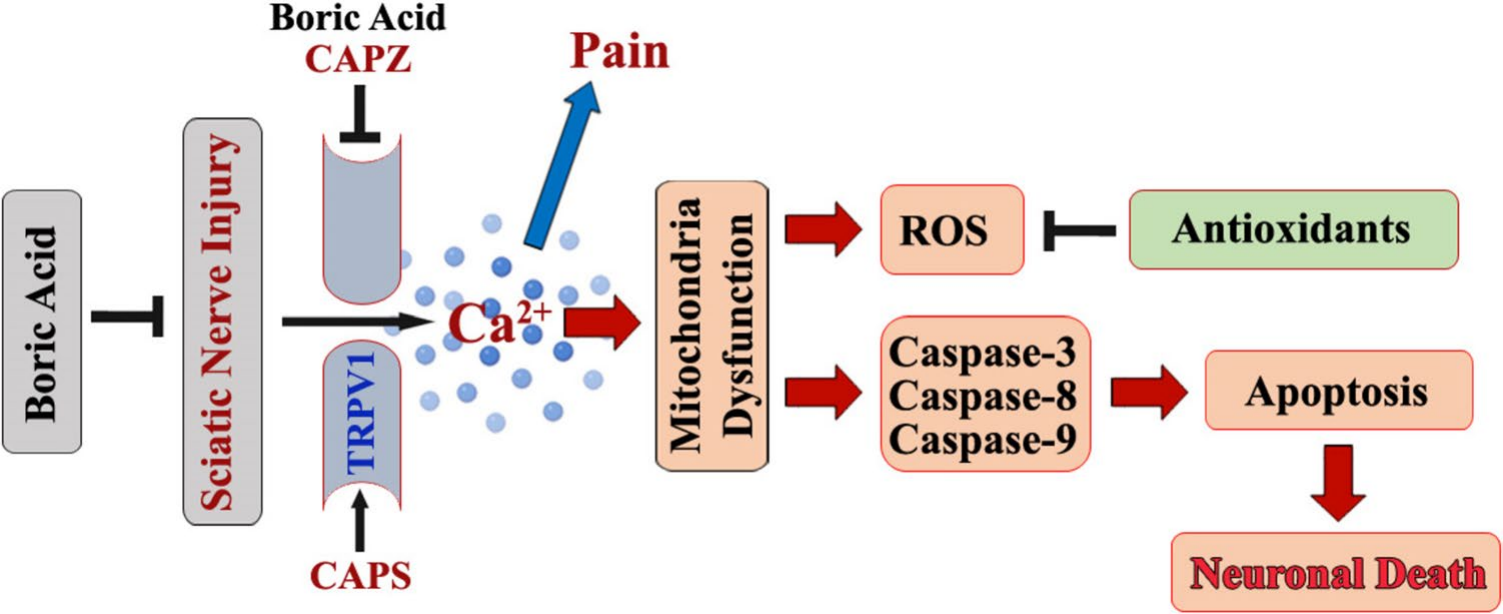
Boric acid inhibits the TRPV1 channel in mice, reducing oxidative stress, apoptosis, and pain induced by SNI. Capsazepine (CAPZ) inhibits the TRPV1 channel, whereas reactive oxygen species (ROS) and capsaicin (CAPS) activate it. Mice with sciatic nerve crashes may experience increased ROS release, pain, and apoptosis induction. This increases Ca^2+^ levels by upregulating TRPV1 while downregulating antioxidants. The buildup of intracellular Ca^2+^ caused mitochondrial dysfunction, which in turn caused additional membrane disruption and the release of proteins that cause apoptosis, including caspases −3, −8, and −9. Because of its anti-pain, anti-apoptotic, and antioxidant qualities, boric acid injection suppresses TRPV1. Boric acid therefore appears to be a promising therapeutic antioxidant for improving the pain, apoptosis, and ROS that SNI causes in mice

In conclusion, elevated Ca^2+^ signaling and mROS pathways caused by SNI-induced TRPV1 stimulation led to pain. By blocking TRPV1 in the sciatic nerve, on the other hand, the BoA injections modulated the negative oxidative, apoptotic, and pain consequences of SNI (Fig. 7). The results of this study demonstrate the therapeutic advantages of BoA as a neuroprotective agent and neuronal signaling recovery agent for peripheral nerve injury by inhibiting TRPV1. Therefore, the BoA may be a useful novel treatment for oxidative stress, apoptosis, and pain produced by SNI by modulating TRPV1.

## Data Availability

No datasets were generated or analysed during the current study.
